# A Review on the Extraction, Bioactivity, and Application of Tea Polysaccharides

**DOI:** 10.3390/molecules27154679

**Published:** 2022-07-22

**Authors:** Jianmei Yao, Huifang Liu, Chiyu Ma, Lulu Pu, Wen Yang, Zhiwei Lei

**Affiliations:** Tea Research Institute, Guizhou Academy of Agricultural Sciences, Huaxi District, Guiyang 550006, China; yjm5055@163.com (J.Y.); 18300865026@163.com (H.L.); chiyuma81@163.com (C.M.); rxl9781@126.com (L.P.); yangwen3409@126.com (W.Y.)

**Keywords:** Tea Polysaccharides, extraction, bioactivity, antioxidant activity

## Abstract

Tea is a non-alcoholic drink containing various active ingredients, including tea polysaccharides (TPSs). TPSs have various biological activities, such as antioxidant, anti-tumor, hypoglycemic, and anti-cancer activities. However, TPSs have a complex composition, which significantly limits the extraction and isolation methods, thus limiting their application. This paper provides insight into the composition, methodological techniques for isolation and extraction of the components, biological activities, and functions of TPSs, as well as their application prospects.

## 1. Introduction

Tea is an important cash crop with a long history of medicinal and dietary benefits, especially in Asian countries, such as China, Thailand, Japan, and India [1,2]. It has various biological functions, including antibacterial, antioxidant, anti-cancer, hypoglycemic, and hypolipidemic [3,4,5]. These biological activities are derived from a variety of chemical components in tea leaves, including polyphenols (TPPs) (catechins, theaflavins, theanines, and other flavonoids), polysaccharides (TPSs), alkaloids (caffeine, theobromine, and theophylline), proteins, lipids, and inorganic elements (selenium, iron, and manganese) [6,7,8,9,10,11]. Polysaccharides have garnered significant interest due to their excellent activity in these chemical compositions [12,13,14].

Tea leaves contain about 1.5–13% of TPS [15,16]. TPSs contain 2–10 monosaccharides, such as glucose, rhamnose, arabinose, mannose, ribose, xylose, galactose, fucose, galacturonic acid, and glucuronic acid, contributing to their large molecular weight range. TPSs and proteins have a similar molecular structure. Proteins contain multiple monosaccharide units linked by glycosidic bonds. There are several TPSs, due to the different monosaccharide linkages [16,17]. As a result, TPSs have various biological activities, such as antioxidant, anti-tumor, anti-diabetic, anti-fatigue, anticoagulant, anti-obesity, hypoglycemic, and immunomodulatory activities [18,19,20,21,22,23]. The complex composition of TPSs affects the isolation and extraction methods, thus limiting the biological activity and application of TPSs. This paper summarizes the extraction methods (aqueous alcoholic precipitation and ultrasonic extraction method), biological activities (antioxidant, anti-diabetic, anti-tumor, and immunomodulatory activities), biological functions (regulation of lipid metabolism, antioxidant, alleviation of blood glucose and lipids), and potential applications (food preservation and drug carriers) of TPSs.

## 2. Components of TPS

**Figure 1:**
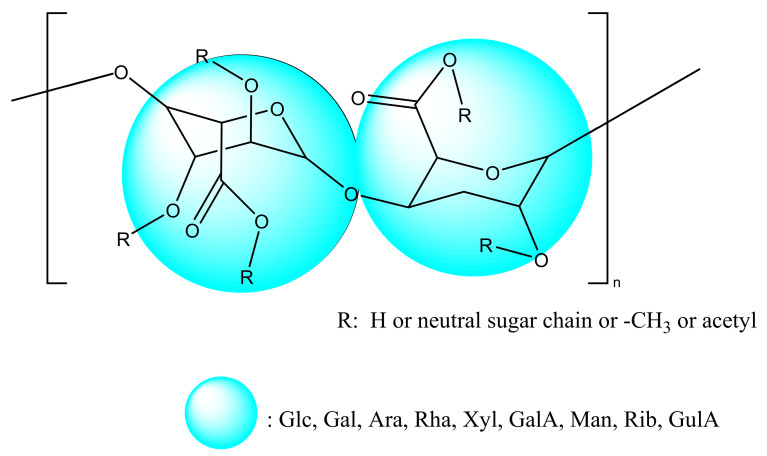
Possible chemical structures of TPS.

TPSs are non-starch protein-bound acidic polysaccharides containing 44.2% neutral sugar, 43.1% glyoxylate, and 3.5% protein. TPSs mainly contain monosaccharides, such as glucose (Glc), galactose (Gal), arabinose (Ara), rhamnose (Rha), xylose (Xyl), galacturonic acid (GalA), mannose (Man), ribose (Rib), and glucuronide (GulA) [24] (Figure 1).

TPSs are also bioactive components of tea with excellent activity, and their crude forms are extracted via simple processing methods. Various processing methods alter the fractions of crude TPSs [25]. Two crude TPSs, TPS1 and TPS2, can be obtained when the aqueous extract of green tea is precipitated with 40% and 70% ethanol, respectively. TPS1 can be separated into two water-soluble TPS1–2a and TPS1–2b with different activities via gel permeation. TPS1–2a and TPS1–2b contain 1,4-linked GalA residues (molecular weight: 20 kDa) and high galacturonic acid (HG) pectin composition with different proportions of carboxymethyl (28.4% and 26.1%, respectively) [26]. The crude water-soluble TPSs can be separated into five fractions (A, B, C, D, and E) via anion-exchange chromatography. Fraction C has the highest inhibitory activity against glucokinase and can be further separated into two tea polysaccharides (C-1 and C-2) via gel chromatography. C-1 is an acidic polysaccharide without protein, mainly composed of rhamnose, arabinose, mannose, glucose, and galactose in a molar ratio of 12.57:22.95:4:39.34:20.77 [27].

TPSs can be divided into neutral polysaccharides (NTPSs) and acidic polysaccharides (ATPSs). NTPSs contain 82.7% of total sugars, of which 12.9% consists of glyoxylates, while ATPSs contain 85.5% of total sugars, of which 39.8% consists of glyoxylates. Gal (67.6%) is the main sugar composition in NTPSs, while Ara, Rha, GalA, and Gal are the main sugar compositions in ATPSs [28]. However, some ATPSs contain nucleic acids [29]. TPSs from some tea leaves also contain Fe, Mg, Zn, Se, and some rare earth elements (REEs), including La, Ce, and Nd [9,10,11].

## 3. Extractions of TPSs

The variation in chemical composition and biological activity of TPSs may be due to the preparation methods and raw materials (Table 1). Various tea plant parts have different TPS contents, compositions, and biological activities. The tea polysaccharide content in tea flowers (TFPS) is 5.24% [30], the tea polysaccharide content in tea leaves (TLPS) is 3.64% [31], and the tea polysaccharide content in tea fruit peel (TFPPS) is 4.98% [32]. In a word, TPS content is highest in tea flowers, followed by tea pericarp, and least in tea leaves. TFPS includes TFPS-1 and TFPS-2. TFPS-1 consists of Glc:Xyl:Rha:Gal in a ratio of 1.0:1.2:0.81:0.98. TFPS-2 contains Glc:Xyl:Rha:Ara in a ratio of 1.0:0.76:2.3:2.3 [30]. The monosaccharides of TLPS are composed of Ara, Xyl, Fuc, Glc, and Gal [31]. TPSs from tea seeds (TSPS) consist of rhamnose, xylose, arabinose, glucose and galactose, GalA, and GulA [33]. TFPPS contains the highest protein content (14.25%) and uronic acid (68.96%), and it also contains seven monosaccharides (rhamnose, mannose, glucose, galactose, arabinose, xylose, and fucose) with different molar ratios [32]. It can be found that different TPSs are obtained by the same extraction method from different parts of tea plants, and their activities are also different. TFPS has antioxidant activity, TLPS has anti-diabetic activity, TSPS has anti-tumor activity, and TFPPS has antioxidant and α-glucosidase inhibitory activities. Different tea polysaccharides are obtained through different extraction methods, and their activities are also different. The two crude tea polysaccharides (TPS1 and TPS2) can be obtained from green tea leaves by water extraction [26]. TPSs coded as TPS-1, TPS-2, TPS-3, and TPS-4 can be obtained from green tea using DEAE-cellulose column extraction [34]. TPS-FC can be obtained from green tea using anion-exchange chromatography [27].

## 4. Bioactivity

Several functional experiments have shown that TPSs have biological activities, including immunomodulatory, anti-tumor, antioxidant, and anti-diabetic activities.

### 4.1. Immunomodulatory Activity

Intracellular polysaccharides (IPSs) isolated from fermented Fuzhuan brick tea (FBT) have immunomodulatory activity in vitro. Specifically, IPSs enhance the immune function of cyclophosphamide (Cy)-induced immunosuppressed mice in vivo by improving immune organ index and immunoglobulins. IPSs also attenuate Cy-induced intestinal barrier damage and promote the expression of tight junction protein and mucin, thus enhancing intestinal barrier function [41]. Cao et al. found that a holistic polysaccharide marker (HPM) enhances the immunomodulatory capacity of the body by increasing the enrichment of splenocytes, the secretion of IL-2, and the toxic activity of NK cells [42]. Ren et al. extracted an acidic polysaccharide (GPTP-3) from gibberellic acid tea and found that GPTP-3 can significantly promote the secretion of macrophage-associated proteins and the immunomodulatory function of cells in mice [43]. Sun et al. extracted polysaccharides from fu brick tea (FBTPs) using aqueous- and alkali-assisted extraction methods. They showed that alkaline-extracted FBTPs (A-FBTPs) have a stronger ability to promote macrophage phagocytosis, acid phosphatase activity, and nitric oxide (NO) secretion in vitro than water-extracted FBTPs (W-FBTPs). In vivo studies have shown that A-FBTPs can induce strong immunomodulatory activity in Cytoxan (CTX)-induced immunosuppressed mice by enhancing the physical characteristics, antioxidant activity, immune response, and intestinal mucosal barrier [44]. Wang et al. conducted immunological tests on two crude TPSs (TPS1 and TPS2) extracted from green tea and showed that TPS-1 can significantly increase the phagocytosis of HL-60 cells, thus enhancing the immunomodulatory activity [26]. Yang et al. isolated crude *Ganoderma lucidum* polysaccharide (LLPs) from samples of *L. lucidus* Turcz herbal tea and evaluated the systemic immunomodulatory activity of LLPs in mice. They found that LLPs can exhibit an overall synergistic stimulatory effect on specific and non-specific immune functions in mice. Moreover, medium and high doses of LLPs significantly increased thymic and splenic organ indices (*p* < 0.05). This finding suggests that polysaccharides extracted from *Ganoderma lucidum* tea can enhance the immune system and may be considered a biological response regulator [45].

### 4.2. Anti-Tumor Activity

Wei et al. investigated the biological activity of tea seed polysaccharides (TSPSs) and showed that TSPSs can significantly inhibit the growth of K562 cells. TSPSs at 50 μg/mL showed the highest inhibitory activity with an inhibition ratio of more than 38.44 ± 2.22% (*p* < 0.01). Higher concentrations (100, 200, and 400 μg/mL) of TSPSs had a stronger proliferative effect on lymphocytes [32]. Yang et al. found that green tea polysaccharides (GTPs) induce anti-tumor effects on PC-3 cells by increasing DAB2 protein expression and inactivating AKT and ERK1/2 signaling pathways. Further mechanistic studies revealed that GTP increases the protein and mRNA levels of DAB2 at all three concentrations [19]. Gao et al. extracted five functional polysaccharides subfractions from Zhongcha 108 using hydrothermal extraction and purified the subfractions via DEAE-52 column chromatography. The tea functional polysaccharides mainly consisted of acidic polysaccharides (containing glucuronic acid) and neutral polysaccharides (composed of rhamnose, galactose, and glucose). The acidic polysaccharides could not inhibit HeLa cell proliferation, while neutral polysaccharides could significantly inhibit HeLa cell proliferation. These results indicate that the TPSs from Zhongcha 108 have certain anti-cancer activity [10]. Cheng et al. obtained a novel heteropolysaccharide (CSP-W-2) from the fruit of Chaenomeles Speciosa (Sweet) Nakai, which could inhibit the growth of HepG2 by enhancing nuclear contraction and apoptosis. Liu et al. extracted a water-soluble homogeneous polysaccharide (DTP-1) from dark brick tea, which could effectively inhibit cancer cell proliferation, induce apoptosis, and suppress migration. However, DTP-1 did not affect normal cells [46]. Park et al. treated peritoneal macrophages of mice with purified polysaccharide GTE-II from mature leaves of green tea and found that GTE-II can increase cytokine production in macrophages and inhibit the growth and metastasis of tumors, indicating that GTE-II has anti-tumor effects [47]. Yang et al. isolated homogeneous polysaccharide (GTP) from green tea (molecular weight: 7.0 × 10^4^ Da) and showed that GTP could inhibit the growth of prostate cancer (PC)-3 cells by inducing apoptosis [20]. Cheng et al. evaluated the in vitro anti-tumor activity of selenium-containing tea polysaccharide (Se-TPS) from selenium-enriched tea using MTT and LDH assays and showed that Se-TPS can significantly inhibit the proliferation of sarcoma 180 (S-180) in a dose-dependent manner [48]. Liu et al. investigated the anti-tumor activity of TPSs isolated from tea leaves against colitis-associated cancer (CAC) and showed that TPSs can significantly reduce tumor incidence and size and inhibit pro-inflammatory cell infiltration and secretion of pro-inflammatory cytokines by balancing the cellular microenvironment. These TPSs could also attenuate CAC proliferation by inhibiting the expression of the IL-6/STAT3 pathway and downstream genes [49].

### 4.3. Antioxidant Activity

The slow removal or excessive production of reactive oxygen species can attack the body’s cells, thus accelerating aging and causing various diseases based on the free radical theory [50]. Han et al. screened the biological and biophysical effects of water-soluble polysaccharides from tea tree flowers (TFPs) and showed that TFPs have significant antioxidant activity in scavenging ROS compared with three commonly used antioxidants. This result suggests that TFPs can be used to treat diseases related to ROS and oxidative damage. TFPs could also be a potential antioxidant for LPO prevention and hepatoprotection [51]. Wang et al. found that ultrasound irradiation can affect the structural properties and antioxidant activity of different yellow TPSs. Ultrasound treatment can degrade polysaccharides without changing the main chemical composition of monosaccharides. Ultrasound irradiation can also increase the free radical scavenging activity of yellow TPS. These results suggest that the alteration of the spatial structures of yellow TPSs can enhance their antioxidant activity [52]. Yang et al. assessed the antioxidant activities of crude TPS and two TPSs (TPS-1 and TPS-2) extracted from green brick tea (QZBT). Using 2,2-diphenyl-1-picryl-hydrazyl (DPPH) and 2,2-azinobis-(3-ethylbenzthiazoline-6-sulfonate) (ABTS) radical scavenging assays, and ferric reducing activity capacity (FRAP) and oxygen radical absorbance capacity (ORAC) assays, Yang et al. showed that the antioxidant activities of the three tea polysaccharides (CTPS, TPS-1, and TPS-2) are concentration-dependent. TPS-2 had a significantly higher antioxidant activity than CTPS and TPS 1 [18]. Fan et al. examined the antioxidant activities of total tea polysaccharide (TTPS), neutral tea polysaccharide (TPSI), and acidic tea polysaccharide (TPSII) extracted from two tea tree cuttings, and showed that TTPS, TPSI, and TPSII have different in vitro antioxidant activities (DPPH, -OH, ABTS free radical scavenging activity and reducing power) and inhibition of α-glucosidase [53]. Guo et al. extracted TPSs from Huaguoshan Yunwu tea and showed that they have high scavenging activity against DPPH, hydroxyl, and superoxide anion radicals and strong reducing and total antioxidant capacity [54]. Xu et al. extracted three acidic TPSs (PTPS-1, PTPS-3, PTPS-5) from Pu’er tea with different fermentation levels and showed that they have good antioxidant activity. Their oxidative activity and α-glucosidase inhibitory activity gradually increased with fermentation level (PTPS-5 > PTPS-3 > PTPS-1) [55]. Xin et al. investigated the effect of different microwave powers on the antioxidant properties (superoxide radicals) of sea buckthorn TPS and showed that 250 W microwave treatment can effectively release and activate the active components in TPS and improve the antioxidant activity of fermented tea [56]. Li et al. investigated the chemical composition and antioxidant activity of Yin Shan Yun Wu TPS and showed that GTPs have a molecular weight of 9.69 × 104 Da and consists of rhamnose, arabinose, xylose, mannose, glucose, and galactose (molar ratio; 11.4:26.1:1.9:3:30.7:26.8). GTPs could also significantly scavenge DPPH radicals, hydroxyl radicals, and superoxide radicals and enhance the in vitro iron reduction capacity [57]. Yuan et al. investigated the antioxidant activity and restorative effects of green TPSs (TPS0, TPS1, TPS2, TPS3) with different molecular weights (Mw) on damaged human proximal renal tubular epithelial cells (HK-2), and showed that the four TPSs have free radical scavenging activity and reducing ability. TPS2 (moderate Mw) had the strongest antioxidant activity [58]. Qin et al. showed that the content and antioxidant activity of TPSs are higher in pine-shaded moonflower (PYR) than in golden-edge moonflower (JBR) and sunflower water moonflower (KSR) [59]. Zheng et al. also showed that TPSs from Ya’an Tibetan tea have high antioxidant activity, mainly in the form of high elimination activity against DPPH and strong reducing power [60].

Yuan et al. showed that the combination of selenium-enriched green tea polysaccharide (Se-GTP) and turkey polysaccharide (HJP) can significantly enhance glutathione peroxidase (GPx) and superoxide dismutase (SOD) activities and reduce malondialdehyde (MDA) levels in mice [61]. Han et al. showed that water-soluble polysaccharide (TFP-1) from *Camellia sinensis* can protect against bromobenzene-induced hepatic lipid peroxidation in mice by increasing superoxide dismutase activity and total antioxidant capacity, and also significantly attenuate malondialdehyde content in a dose-dependent manner [53]. Fan et al. assessed the effect of the purity of TPSs on their antioxidant activity, and showed that the higher the purity of TPS, the lower the antioxidant capacity. TPSs are polysaccharide–protein complexes, and their purification can remove the unstable binding protein from the complex, thus reducing the antioxidant activity [62]. Liu et al. evaluated the cell-based in vitro antioxidant effect of Tianshan green tea polysaccharide (TSPS) and showed that TSPSs have excellent antioxidant capacity against DPPH radicals, hydroxyl radicals, and ABTS radicals and enhanced iron reduction capacity (FRAP) [63]. Wang et al. also investigated the effects of ultrasonic irradiation on the structural characteristics and antioxidant properties of yellow TPS with different molecular weights (Mw). Ultrasound treatment degraded polysaccharide without changing the main chemical composition of monosaccharides, and also altered the free radical scavenging activity of yellow TPA. As a result, the ultrasonically degraded yellow TPSs have a stronger antioxidant capacity [54]. Chen et al. isolated crude TPS and four CTPS fractions (TPF30, TPF50, TPF70, and TPF90) from green tea and compared their antioxidant activities. TPF90 had significant DPPH-scavenging activity and the highest inhibitory effect on hydroxyl radicals, reducing power, and chelating activity, showing stronger antioxidant capacity than crude TPS [64]. Shu et al. explored the antioxidant activity of green tea powders (GTPs) with different particle sizes using a simulated in vitro gastrointestinal digestion model and showed that the particle size significantly affects the antioxidant activity of the polysaccharides. However, this could be improved by appropriately reducing the particle size [65].

### 4.4. Anti-Diabetic Activity

TPSs in coarse old tea leaves are widely used to treat diabetes in Chinese and Japanese folklore [31]. The hypoglycemic effects of TPSs have attracted much attention since the incidence of diabetes is increasing worldwide. Guo et al. extracted tea polysaccharides (TPSs) from 12 typical Chinese teas (*Camellia sinensis*) via an aqueous extraction method and evaluated their anti-diabetic activity. The TPSs exhibited significant antioxidant and anti-diabetic (α-glucosidase inhibition and anticoagulant) activities. Particularly, Pu’er TPS had good anti-diabetic activity [66]. Wang et al. found that TPSs (at high concentrations) can improve the vascular system and reduce the lipid content in diabetic patients [31]. Wu et al. prepared bioactive acidic TPSs from the yellow leaves of Wuyi rock tea using DEAE-52 and Superdex-200 columns and evaluated the hypoglycemic effect of acidic TPSs using streptozotocin-induced type 2 diabetic rats. Acidic TPSs could improve plasma and hepatic lipid metabolism, suggesting that TPSs can improve glucose metabolism disorders and gut flora in hyperglycemic rats [67]. Chung et al. used an in vitro digestion model of Caco-2 cells to investigate the combined effect of green tea extract (GTE) and crude green tea polysaccharides (CTPs) on inhibiting glucose transport after digestion of rice starch. They discovered that GTE + CTP can significantly inhibit glucose transport from the digestive tract to Caco-2 cells when compared to the control group. These results indicate that rice consumption with GTE and green TPS can reduce postprandial glucose levels [68]. Mao et al. investigated the hypolipidemic effect of Chinese six-bubble tea polysaccharide (CLTPS) and showed that CLTPS can significantly improve the levels of lipid profile, lipid oxidation, and antioxidant enzyme activities in a dose-dependent manner. CLTPSs have a significant protective effect against high-fat-diet-induced dyslipidemia [69]. Deng et al. extracted TPSs from dog bone brain tea and showed that the TPSs have a strong inhibitory effect on α-amylase and α-glucosidase. Moreover, their hypoglycemic activities were concentration-dependent [70]. Guo et al. evaluated the anti-diabetic activity of TPSs extracted from 12 representative edible Chinese teas (*Camellia sinensis* L.) and showed that black TPSs have stronger α-glucosidase inhibitory and anti-diabetic activities than the other selected tea polysaccharides [66]. Luo et al. isolated polysaccharides from guava leaves (GLPs) and showed that GLPs can significantly reduce fasting glucose, total cholesterol, total triglycerides, glycated serum protein, creatinine, and malondialdehyde in Streptozotocin-induced diabetic mice [71]. Le et al. extracted and purified *Nelumbo nucifera* polysaccharide (NNP-2) from lotus seed tea and investigated its anti-diabetic activity and the mechanism of regulating glucose metabolism in HepG2 cells. They found that NNP-2 has biological activity against α-glucosidase (IC_50_ = 97.32 µg/mL) and could improve intracellular glucose levels by regulating the IRS1/PI3 K/Akt pathway in HepG2 cells [72].

## 5. Function

### 5.1. Strengthened Immune Function

Bai et al. investigated the effects of crude polysaccharides from Fuzhuan brick tea (CFBTPS) and purified fractions (FBTPS-3) extracted from porcupine tea on the regulation of immune function and intestinal microbiota in mice, and found the following results: CFBTPS and FBTPS-3 could restore the levels of various physiological indicators, such as body weight, diet, immunoglobulins, and immune organ indices in mice. CFBTPS and FBTPS-3 could also repair intestinal damage and regulate the intestinal microbiota. Moreover, FBTPS could regulate immune function in mice [73].

### 5.2. Regulation of Sugar and Lipids in the Blood

TPSs have a hypoglycemic effect. Numerous studies have demonstrated that TPSs have significant therapeutic effects on diabetes. Tang et al. investigated the biological activities of two fractions (JSP-1 and JSP-2) of water-soluble polysaccharides (JSPs) extracted from Eton tea and showed that JSP-1 and JSP-2 could scavenge DPPH and hydroxyl radicals and protect pancreatic islet cells. These results indicate the antioxidant and hypoglycemic activities of JSP-1 and JSP. However, JSP-1 had stronger antioxidant and hypoglycemic activities than JSP-2 [74].

### 5.3. Lipid Metabolism Regulation

Obesity has become a global health problem due to changes in diet and living habits. Several studies have shown that tea can regulate lipid metabolism. Wu et al. found that polysaccharide extract can reduce the weight of rats with hfD-induced obesity, improve body composition, and increase the content of fatty acids in feces, thus preventing hfD-induced obesity. They also explored the mechanism of polysaccharide extracts in regulating lipid metabolism via gene chip array and found that polysaccharide extracts mainly affect gene expression of the lipid metabolism pathway, thus causing biochemical changes, such as increasing fecal fatty acid content and decreasing body fatty acid content [75]. Xu et al. found that green tea extracts, especially polysaccharides, induce a strong inhibitory effect on fat in rats by reducing serum leptin and inhibiting the absorption of fatty acids. They also found that polyphenols and polysaccharides have a synergistic effect on reducing the level of serum leptin and anti-inflammatory activity [76].

### 5.4. Other Functions

Chen et al. investigated the therapeutic effect of TPSs on colitis in mice. They showed that TPSs could alleviate colitis by restoring various physical indices (body weight, colon length, and disease activity index) and promoting intestinal barrier function [77].

## 6. Application

### 6.1. Drug Carriers

Polysaccharides have natural advantages in biomedical applications due to the unique multifunctional groups on their structure. Polysaccharides can be modified to polysaccharide-based nanoparticles with various structures via simple chemical or biochemical methods. Moreover, polysaccharides can be used as a carrier of different drugs since their structures have good biocompatibility and degradability and cannot easily leak into the blood, thus achieving longer blood circulation [78,79,80,81].

For example, polysaccharide-gel-coated drugs can be orally taken to specific sites to deliver their effects [82].

Wu et al. designed and synthesized polysaccharide-based porous microspheres (PPMs) via the inverse emulsion polymerization method and evaluated their drug delivery capabilities and functionalities. The PPMs loaded with mitomycin-C (MMC) could promote the sustained release of MMC at the target site, thus reducing the toxic effects on normal tissues and exhibiting stronger tumor-suppressive effects [83].

### 6.2. Packaging Materials for Food Products

Common plastic products are environmental pollutants. As a result, polysaccharides have attracted much attention because they can be developed into biodegradable materials and coatings [84,85]. Fruits and vegetables can be preserved with edible films and coatings of starch-based polysaccharides, thereby prolonging shelf life and decreasing water loss [86]. Kumar et al. found that natural products (polysaccharides, proteins, and lipids) mixed with plasticizers (glycerol and glycols) and surfactants can form substances that can be made into edible films. These films can prevent or inhibit the release of organic vapors (solvents and aromas), water vapor, solutes, and gases (carbon dioxide, oxygen, and nitrogen), thus preventing food degradation and extending shelf life [84,85]. Zhu et al. also found that a composite polysaccharide film made by mixing polysaccharides and polyphenols can inhibit the spread of SARS-CoV-2 through the food supply chain [87].

## 7. Prospect

The variation in chemical composition and biological activity of TPSs may be due to the different preparation methods and raw tea samples. Some common TPS preparation methods have low yields and alter the active components during extraction. Therefore, an efficient, standardized, and simple method should be established for purifying TPSs from various tea leaves to obtain chemically stable TPS products.

Various types of polysaccharide drugs have been introduced into clinical practice in the past 20 years for the treatment of various diseases in China and worldwide. About 30 types of polysaccharide drugs with anti-tumor, anti-infection, anti-rheumatism, anti-peptic ulcer, and immune functions are currently used in clinics. The drugs may be in the form of polysaccharide drugs, anti-radiation drinks, chewing gums, and various health products. TPSs can be added to coffee, beer, and other foods to make unique functional foods. TPSs can also act as food additives in beverages, pastries, and oral liquids. However, in-depth research on TPSs is necessary to discover more valuable aspects, including the auxiliary treatment of diabetes.

## Figures and Tables

**Table 1:** Compositions and contents of TPSs in different parts of tea plant.

Resources	Extraction Methods	Main Components	Bioactivities	References
Tea flower	Boiling water extraction and ethanol precipitation	TPSs include TFPS-1 and TFPS-2; TFPS-1 consists of Glc:Xyl:Rha:Gal in a ratio of 1.0:1.2:0.81:0.98; TFPS-2 contains Glc:Xyl:Rha:Ara in a ratio of 1.0:0.76:2.3:2.3	Antioxidant activity	Han et al. [30]
Tea leaves		The monosaccharides of the saccharide in TPSs are composed of Ara, Xyl, Fuc, Glc, and Gal	Anti-diabetic activity	Wang et al. [31]
Tea seed	Water extraction	TSPSs consist of rhamnose, xylose, arabinose, glucose and galactose, GalA, and GulA	Anti-tumor activity	Wei et al. [33]
Tea fruit peel	Water extraction	TFPP-Crude contains the highest protein content (14.25%) and uronic acid (68.96%). TFPP-60 has the highest content of neutral sugar (23.00%) and the lowest content of uronic acid (46.42%). It also contains seven monosaccharides (rhamnose, mannose, glucose, galactose, arabinose, xylose, and fucose) with different molar ratios	Antioxidant activity and α-glucosidase inhibitory activity	Wang et al. [32]
Green tea, oolong tea, and black tea	Boiling water extraction and ethanol precipitation	Three polysaccharides (TTPS, FTPS, and DTPS) can be isolated from these three teas. The two crude tea polysaccharides (TPS1 and TPS2) can be obtained from green tea leaves. Three polysaccharide-rich fractions, GTPs, OTPs, and BTPs, can be isolated from green tea, oolong tea, and black tea	Antioxidant activity and alpha-glucosidase inhibitory effect, phagocytosis effect. Immunomodulatory activity, hypoglycemic activities	Wang et al., Chen et al., Yang et al. [26,35,36,37]
Green tea	DEAE-cellulose column	TPSs coded as TPS-1, TPS-2, TPS-3, and TPS-4		Guo et al. [34]
Green tea	RP-C18 column	TPSs consist of mannose, ribose, rhamnose, glucuronic acid, galacturonic acid, glucose, xylose, galactose, and arabinose		Lv et al. [24]
Green tea	Anion-exchange chromatography	TPS-FC	Glucokinase-stimulating activity	Wang et al. [27]
Green tea	Alcohol precipitation	TPS	Regulation of sugar and lipids in the blood	Oh et al., Koh, et al. [38,39]
Green tea, oolong tea, and black tea	Ultrafiltration method	TPS	Antioxidant activities, α-glucosidase inhibitory effect	Xu et al. [40]

## References

[1] Xiao J.B., Jiang H.X. (2015). A review on the structure-function relationship aspect of polysaccharides from tea materials. Crit. Rev. Food Sci. Nutr..

[2] Zheng X.Q., Li Q.S., Xiang L.P., Liang Y.R. (2016). Recent advances in volatiles of teas. Molecules.

[3] Liu Y.C., Li X.Y., Shen L. (2020). Modulation effect of tea consumption on gut microbiota. Appl. Microbiol. Biotechnol..

[4] Sanlier N., Gokcen B.B., Altug M. (2018). Tea consumption and disease correlations. Trends Food Sci. Technol..

[5] Yang C.S., Wang H., Sheridan Z.P. (2018). Studies on prevention of obesity, metabolic syndrome, diabetes, cardiovascular diseases and cancer by tea. J. Food Drug Anal..

[6] Chen X.Q., Du Y., Wu L., Xie J.C., Chen X.L., Hu B.B., Wu Z.Q., Yao Q.F., Li Q. (2019). Effects of tea-polysaccharide conjugates and metal ions on precipitate formation by epigallocatechin gallate and caffeine, the key components of green tea infusion. J. Agric. Food Chem..

[7] Cheng M., Zhang X., Guo X.J., Wu Z.F., Weng P.F. (2017). The interaction effect and mechanism between tea polyphenols and intestinal microbiota: Role in human health. J. Food Biochem..

[8] Hu X.Y., Wang Y.M., Zhang L.L., Xu M. (2020). Construction of self-assembled polyelectrolyte complex hydrogel based on oppositely charged polysaccharides for sustained delivery of green tea polyphenols. Food Chem..

[9] Puligundla P., Mok C., Ko S., Liang J., Recharla N. (2017). Nanotechnological approaches to enhance the bioavailability and therapeutic efficacy of green tea polyphenols. J. Funct. Foods.

[10] Gao Y.F., Zhou Y.B., Zhang Q., Zhang K., Peng P., Chen L.C., Xiao B. (2017). Hydrothermal extraction, structural characterization, and inhibition HeLa cells proliferation of functional polysaccharides from Chinese tea Zhongcha 108. J. Funct. Foods.

[11] Nie S.P., Xie M.Y. (2011). A review on the isolation and structure of tea polysaccharides and their bioactivities. Food Hydrocoll..

[12] Li F., Wei Y.L., Liang L., Huang L.L., Yu G.Y., Li Q.H. (2021). A novel low-molecular-mass pumpkin polysaccharide: Structural characterization, antioxidant activity, and hypoglycemic potential. Carbohydr. Polym..

[13] Liu H., Sun X.Y., Wang F.X., Ouyang J.M. (2020). Regulation on calcium oxalate crystallization and protection on HK-2 cells of tea polysaccharides with different molecular weights. Oxid. Med. Cell. Longev..

[14] Ma Z.X., Liu J., Liu Y.C., Zheng X.J., Tang K.Y. (2021). Green synthesis of silver nanoparticles using soluble soybean polysaccharide and their application in antibacterial coatings. Int. J. Biol. Macromol..

[15] Nie S., Cui S.W., Xie M. (2018). Chapter 7—Tea polysaccharide. Bioactive Polysaccharides.

[16] Hu T., Wu P., Zhan J.F., Wang W.X., Shen J.F., Ho C.T., Li S.M. (2021). Influencing factors on the physicochemical characteristics of tea polysaccharides. Molecules.

[17] Chen G.J., Yuan Q.X., Saeeduddin M., Ou S.Y., Zeng X.X., Ye H. (2016). Recent advances in tea polysaccharides: Extraction, purification, physicochemical characterization and bioactivities. Carbohydr. Polym..

[18] Yang X.H., Huang M.J., Qin C.Q., Lv B.Y., Mao Q.L., Liu Z.H. (2017). Structural characterization and evaluation of the antioxidant activities of polysaccharides extracted from Qingzhuan brick tea. Int. J. Biol. Macromol..

[19] Yang K., Li Y.W., Gao Z.Y., Xiao W., Li T.Q., Song W., Zheng J., Chen H., Chen G.H., Zou H.Y. (2019). MiR-93 functions as a tumor promoter in prostate cancer by targeting disabled homolog 2 (DAB2) and an antitumor polysaccharide from green tea (*Camellia sinensis*) on their expression. Int. J. Biol. Macromol..

[20] Yang K., Gao Z.Y., Li T.Q., Song W., Xiao W., Zheng J., Chen H., Chen G.H., Zou H.Y. (2019). Anti-tumor activity and the mechanism of a green tea (*Camellia sinensis*) polysaccharide on prostate cancer. Int. J. Biol. Macromol..

[21] Zhu J.X., Chen Z.Y., Zhou H., Yu C., Han Z., Shao S.R., Hu X.C., Wei X.L., Wang Y.F. (2020). Effects of extraction methods on physicochemical properties and hypoglycemic activities of polysaccharides from coarse green tea. Glycoconj. J..

[22] Wang H.J., Shi S.S., Bao B., Li X.J., Wang S.C. (2015). Structure characterization of an arabinogalactan from green tea and its anti-diabetic effect. Carbohydr. Polym..

[23] Chen G.J., Bai Y.X., Zeng Z.Q., Peng Y.J., Zhou W.T., Shen W.B., Zeng X.X., Liu Z.H. (2021). Structural characterization and immunostimulatory activity of heteropolysaccharides from Fuzhuan brick tea. J. Agric. Food Chem..

[24] Lv Y., Yang X.B., Zhao Y., Ruan Y., Yang Y., Wang Z.Z. (2009). Separation and quantification of component monosaccharides of the tea polysaccharides from *Gynostemma pentaphyllum* by HPLC with indirect UV detection. Food Chem..

[25] Wang Y.F., Liu Y.Y., Huo J.L., Zhao T.T., Ren J., Wei X.L. (2013). Effect of different drying methods on chemical composition and bioactivity of tea polysaccharides. Int. J. Biol. Macromol..

[26] Wang H.J., Wei G.D., Liu F., Banerjee G., Joshi M., Bligh S.W.A., Shi S.S., Lian H., Fan H.W., Gu X.L. (2014). Characterization of two homogalacturonan pectins with immunomodulatory activity from green tea. Int. J. Mol. Sci..

[27] Wang L., Xia W.S. (2006). Isolation and analysis of a novel acidic polysaccharide with glucokinase-stimulating activity from coarse green tea. J. Food Biochem..

[28] Wang Y.F., Peng Y.H., Wei X.L., Yang Z.W., Xiao J.B., Jin Z.Y. (2010). Sulfation of tea polysaccharides: Synthesis, characterization and hypoglycemic activity. Int. J. Biol. Macromol..

[29] Chen X.Q., Ye Y., Cheng H., Jiang Y.W., Wu Y.L. (2009). Thermal effects on the stability and antioxidant activity of an acid polysaccharide conjugate derived from green tea. J. Agric. Food Chem..

[30] Han Q.A., Yu Q.Y., Shi J.A., Xiong C.Y., Ling Z.J., He P.M. (2011). Structural characterization and antioxidant activities of 2 water-soluble polysaccharide fractions purified from tea (*Camellia sinensis*) flower. J. Food Sci..

[31] Wang D.F., Wang C.H., Li J., Zhao G.W. (2001). Components and activity of polysaccharides from coarse tea. J. Agric. Food Chem..

[32] Wang Y.F., Wang J., Wu J., Xu P., Wang Y.Q., Gao J.J., Hochstetter D. (2014). In vitro antioxidant activity and potential inhibitory action against alpha-glucosidase of polysaccharides from fruit peel of tea (*Camellia sinensis* L.). J. Zhejiang Univ. Sci. B.

[33] Wei X.L., Mao F.F., Cai X., Wang Y.F. (2011). Composition and bioactivity of polysaccharides from tea seeds obtained by water extraction. Int. J. Biol. Macromol..

[34] Guo L., Liang Q., Du X.F. (2011). Effects of molecular characteristics of tea polysaccharide in green tea on glass transitions of potato amylose, amylopectin and their mixtures. Food Hydrocoll..

[35] Wang Y.F., Shao S.H., Xu P., Chen H., Lin-Shiau S.Y., Deng Y.T., Lin J.K. (2012). Fermentation process enhanced production and bioactivities of oolong tea polysaccharides. Food Res. Int..

[36] Chen H.X., Qu Z., Fu L.L., Dong P., Zhang X. (2009). Physicochemical properties and antioxidant capacity of 3 polysaccharides from green tea, oolong tea, and black tea. J. Food Sci..

[37] Yang X.B., Zhao Y., Yang Y., Ruan Y. (2008). Isolation and characterization of immunostimulatory polysaccharide from an herb tea, *Gynostemma pentaphyllum* Makino. J. Agric. Food Chem..

[38] Oh J.H., Lee C.Y., Kim J.E., Kim W.H., Seo J.W., Lim T.G., Lee S.Y., Chung J.O., Hong Y.D., Kim W.G. (2021). Effect of characterized green tea extraction methods and formulations on enzymatic starch hydrolysis and intestinal glucose transport. J. Agric. Food Chem..

[39] Koh G.Y., Chou G.X., Liu Z.J. (2009). Purification of a water extract of Chinese sweet tea plant (*Rubus suavissimus* S. Lee) by alcohol precipitation. J. Agric. Food Chem..

[40] Xu L.L., Chen Y., Chen Z.Q., Gao X.D., Wang C.L., Panichayupakaranant P., Chen H.X. (2020). Ultrafiltration isolation, physicochemical characterization, and antidiabetic activities analysis of polysaccharides from green tea, oolong tea, and black tea. J. Food Sci..

[41] Xie Z.Y., Bai Y.X., Chen G.J., Dong W., Peng Y.J., Xu W.Q., Sun Y., Zeng X.X., Liu Z.H. (2022). Immunomodulatory activity of polysaccharides from the mycelium of *Aspergillus cristatus*, isolated from Fuzhuan brick tea, associated with the regulation of intestinal barrier function and gut microbiota. Food Res. Int..

[42] Cao Z.J., Yip K.M., Jiang Y.G., Ji S.L., Ruan J.Q., Wang C., Chen H.B. (2020). Suitability evaluation on material specifcations and edible methods of Dendrobii Ofcinalis Caulis based on holistic polysaccharide marker. Chin. Med..

[43] Ren D.Y., Zhao Y., Zheng Q., Alim A., Yang X.B. (2019). Immunomodulatory effects of an acidic polysaccharide fraction from herbal *Gynostemma pentaphyllum* tea in RAW264.7 cells. Food Funct..

[44] Sun Y.J., Wang F., Liu Y., An Y.Y., Chang D.W., Wang J.K., Xia F., Liu N., Chen X.F., Cao Y.G. (2022). Comparison of water- and alkali-extracted polysaccharides from Fuzhuan brick tea and their immunomodulatory effects in vitro and in vivo. Food Funct..

[45] Yang X.B., Lv Y., Tian L.M., Zhao Y. (2010). Composition and systemic immune activity of the polysaccharides from an herbal tea (*Lycopus lucidus* Turcz). J. Agric. Food Chem..

[46] Liu M., Gong Z., Liu H., Wang J.H., Wang D., Yang Y.J., Zhong S.A. (2022). Structural characterization and anti-tumor activity in vitro of a water-soluble polysaccharide from dark brick tea. Int. J. Biol. Macromol..

[47] Park H.R., Hwang D.Y., Suh H.J., Yu K.W., Kim T.Y., Shin K.S. (2017). Antitumor and antimetastatic activities of rhamnogalacturonan-II-type polysaccharide isolated from mature leaves of green tea via activation of macrophages and natural killer cells. Int. J. Biol. Macromol..

[48] Cheng L.Z., Chen L., Yang Q.Q., Wang Y.F., Wei X.L. (2018). Antitumor activity of Se-containing tea polysaccharides against sarcoma 180 and comparison with regular tea polysaccharides and Se-yeast. Int. J. Biol. Macromol..

[49] Liu L.Q., Nie S.P., Shen M.Y., Hu J.L., Yu Q., Gong D.M., Xie M.Y. (2018). Tea polysaccharides inhibit colitis-associated colorectal cancer via interleukin-6/STAT3 pathway. J. Agric. Food Chem..

[50] Zhang C.L., Fu L.N., Yang X.Y., Li K., Xin J.H. (2015). Review of the relationship between ROS and cell senescence. Guangzhou Chem. Ind..

[51] Han Q., Xiong C.Y., Shi J., Gao Y., Chen Y.S., Ling Z.J., He P.M. (2012). Isolation, chemical characterization and antioxidant activities of a water-soluble polysaccharide fraction of tea (*Camellia sinensis*) flower. J. Food Biochem..

[52] Wang H.S., Chen J.R., Ren P.F., Zhang Y.W., Onayango S.O. (2021). Ultrasound irradiation alters the spatial structure and improves the antioxidant activity of the yellow tea polysaccharide. Ultrason. Sonochem..

[53] Fan M.H., Sun X., Qian Y.L., Xu Y., Wang D.F., Cao Y.P. (2018). Effects of metal ions in tea polysaccharides on their in vitro antioxidant activity and hypoglycemic activity. Int. J. Biol. Macromol..

[54] Guo L., Guo J.C., Zhu W.C., Jiang X.R. (2016). Optimized synchronous extraction process of tea polyphenols and polysaccharides from Huaguoshan Yunwu tea and their antioxidant Activities. Food Bioprod. Process..

[55] Xu P., Wu J., Zhang Y., Chen H., Wang Y.F. (2014). Physicochemical characterization of puerh tea polysaccharides and their antioxidant and a-glycosidase inhibition. J. Funct. Foods.

[56] Xin Y.H., Zhao S.H., Yang J., Zhang T.D., Zhang J.H., Wang Y. (2021). Effect of microwave-drying on the quality and antioxidant properties of *Ganoderma lucidum* fermented sea-buckthorn tea. Int. J. Food Eng..

[57] Li X., Chen S., Li J.E., Wang N., Liu X., An Q., Ye X.M., Zhao Z.T., Zhao M., Han Y. (2019). Chemical composition and antioxidant activities of polysaccharides from Yingshan cloud mist tea. Oxid. Med. Cell. Longev..

[58] Sun X.Y., Wang J.M., Ouyang J.M., Kuang L. (2018). Antioxidant activities and repair effects on oxidatively damaged HK-2 cells of tea polysaccharides with different molecular weights. Oxid. Med. Cell. Longev..

[59] Qin H., Deng X.Q., Li B.C., Dai W.F., Jiao S.Y., Qin Y., Zhang M. (2018). Volatiles, polysaccharides and total polyphenols in Chinese rose tea infusions and their antioxidant activities. J. Food Process. Preserv..

[60] Zheng Q.R., Li W.F., Zhang H., Gao X.Y., Tan S. (2020). Optimizing synchronous extraction and antioxidant activity evaluation of polyphenols and polysaccharides from Ya’an Tibetan tea (*Camellia sinensis*). Food Sci. Nutr..

[61] Yuan C.F., Li Z.H., Peng F., Xiao F.X., Ren D.M., Xue H., Chen T., Mushtaq G., Kamal M.A. (2015). Combination of selenium-enriched green tea polysaccharides and Huo-ji polysaccharides synergistically enhances antioxidant and immune activity in mice. J. Sci. Food Agric..

[62] Fan M.H., Zhu J.X., Qian Y.L., Yue W., Xu Y., Zhang D.D., Yang Y.Q., Gao X.Y., He H.Y., Wang D.F. (2020). Effect of purity of tea polysaccharides on its antioxidant and hypoglycemic activities. J. Food Biochem..

[63] Liu J.B., Lin J., Huang Z.H., Zheng Q.X., Lin F., Wu L.Y. (2022). Chemical characterization of Tianshan green tea polysaccharides and its protective effects on cell oxidative injury. J. Food Biochem..

[64] Chen P., Zhang Y.J., Zhu L.Y., Jin H., Zhang G.F., Su D.Y., Li J. (2013). Chemical analysis and antioxidant activity in vitro of polysaccharides extracted from lower grade green tea. Adv. J. Food Sci. Technol..

[65] Shu Y., Li J., Yang X.P., Dong X.Y., Wang X.J. (2019). Effect of particle size on the bioaccessibility of polyphenols and polysaccharides in green tea powder and its antioxidant activity after simulated human digestion. J. Food Sci. Technol..

[66] Guo H., Fu M.X., Wu D.T., Zhao Y.X., Li H., Li H.B., Gan R.Y. (2021). Structural characteristics of crude polysaccharides from 12 selected chinese teas, and their antioxidant and anti-diabetic activities. Antioxidants.

[67] Wu Z., Zeng W., Zhang X., Yang J. (2022). Characterization of acidic tea polysaccharides from yellow leaves of wuyi rock tea and their hypoglycemic activity via intestinal flora regulation in rats. Foods.

[68] Chung J.O., Yoo S.H., Lee Y.E., Shin K.S., Yoo S.J., Park S.H., Park T.S., Shim S.M. (2019). Hypoglycemic potential of whole green tea: Water-soluble green tea polysaccharides combined with green tea extract delays digestibility and intestinal glucose transport of rice starch. Food Funct..

[69] Mao Y., Wei B.Y., Teng J.W., Xia N., Zhao M.M., Huang L., Ye Y. (2018). Polysaccharides from Chinese Liupao dark tea and their protective effect against hyperlipidemia. Int. J. Food Sci. Technol..

[70] Deng Q.H., Wang W.J., Zhang Q.F., Chen J.G., Zhou H., Meng W.Y., Li J.G. (2021). Extraction optimization of polysaccharides from Gougunao tea and assessment of the antioxidant and hypoglycemic activities of its fractions in vitro. Bioact. Carbohydr. Diet. Fibre.

[71] Luo Y., Peng B., Wei W.Q., Tian X.F., Wu Z.Q. (2019). Antioxidant and anti-diabetic activities of polysaccharides from guava leaves. Molecules.

[72] Le B., Anh P.T.N., Yang S.H. (2021). Polysaccharide derived from *Nelumbo nucifera* lotus plumule shows potential prebiotic activity and ameliorates insulin resistance in HepG2 cells. Polymers.

[73] Bai Y., Zeng Z., Xie Z., Chen G., Chen D., Sun Y., Zeng X., Liu Z. (2022). Effects of polysaccharides from Fuzhuan brick tea on immune function and gut microbiota of cyclophosphamide-treated mice. J. Nutr. Biochem..

[74] Tang Y., Sheng J., He X., Sun J., Wei Z., Liu G., Li C., Lin B., Li L. (2021). Novel antioxidant and hypoglycemic water-soluble polysaccharides from jasmine tea. Foods.

[75] Wu T., Guo Y., Liu R., Wang K., Zhang M. (2016). Black tea polyphenols and polysaccharides improve body composition, increase fecal fatty acid, and regulate fat metabolism in high-fat diet-induced obese rats. Food Funct..

[76] Xu Y., Zhang M., Wu T., Dai S., Xu J., Zhou Z. (2015). The anti-obesity effect of green tea polysaccharides, polyphenols and caffeine in rats fed with a high-fat diet. Food Funct..

[77] Chen C., Wang H., Hong T., Huang X., Xia S., Zhang Y., Chen X., Zhong Y., Nie S. (2022). Effects of tea polysaccharides in combination with polyphenols on dextran sodium sulfate-induced colitis in mice. Food Chem. X.

[78] Debele T.A., Mekuria S.L., Tsai H.C. (2016). Polysaccharide based nanogels in the drug delivery system: Application as the carrier of pharmaceutical agents. Mater. Sci. Eng. C.

[79] Yang Y., Xu L., Wang J., Meng Q., Zhong S., Gao Y., Cui X. (2022). Recent advances in polysaccharide-based self-healing hydrogels for biomedical applications. Carbohydr. Polym..

[80] Sood A., Gupta A., Agrawal G. (2021). Recent advances in polysaccharides based biomaterials for drug delivery and tissue engineering applications. Carbohydr. Polym. Technol. Appl..

[81] Meng Q., Zhong S., Xu L., Wang J., Zhang Z., Gao Y., Cui X. (2022). Review on design strategies and considerations of polysaccharide-based smart drug delivery systems for cancer therapy. Carbohydr. Polym..

[82] Ghosh T., Mukherjee S. (2022). Cross-linked ionic polysaccharides: Insights from the structure to stimuli-sensitive drug delivery system applications. Asian J. Chem..

[83] Wu Y.B., Zhang J.D., Ni J.W., Yang Z.H., Chen K., Zheng L.C., He Z.F. (2021). Polysaccharide-based lotus seedpod surface-like porous microsphere as an efficient drug carrier for cancer treatment. Cancer Manag. Res..

[84] Cazon P., Velazquez G., Ramirez J.A., Vazquez M. (2017). Polysaccharide-based films and coatings for food packaging: A review. Food Hydrocoll..

[85] Kumar N., Neeraj (2019). Polysaccharide-based component and their relevance in edible film/coating: A review. Nutr. Food Sci..

[86] Sapper M., Chiralt A. (2018). Starch-based coatings for preservation of fruits and vegetables. Coatings.

[87] Zhu F. (2021). Polysaccharide based films and coatings for food packaging: Effect of added polyphenols. Food Chem..

